# Thermosensitive Chitosan-*β*-Glycerophosphate Hydrogels as Targeted Drug Delivery Systems: An Overview on Preparation and Their Applications

**DOI:** 10.1155/2021/6640893

**Published:** 2021-05-05

**Authors:** Pouria Rahmanian-Devin, Vafa Baradaran Rahimi, Vahid Reza Askari

**Affiliations:** ^1^Department of Pharmaceutics, School of Pharmacy, Mashhad University of Medical Sciences, Mashhad, Iran; ^2^Applied Biomedical Research Center, Mashhad University of Medical Sciences, Mashhad, Iran; ^3^Department of Pharmaceutical Sciences in Persian Medicine, School of Persian and Complementary Medicine, Mashhad University of Medical Sciences, Mashhad, Iran; ^4^Department of Persian Medicine, School of Persian and Complementary Medicine, Mashhad University of Medical Sciences, Mashhad, Iran

## Abstract

Today, with the advances in technology and science, more advanced drug delivery formulations are required. One of these new systems is an intelligent hydrogel. These systems are affected by the environment or conditions that become a gel, stay in the circumstance for a certain period, and slowly release the drug. As an advantage, only a lower dose of the drug is required, and it provides less toxicity and minor damage to other tissues. Hydrogels are of different types, including temperature-sensitive, pH-sensitive, ion change-sensitive, and magnetic field-sensitive. In this study, we investigated a kind of temperature-sensitive smart hydrogel, which has a liquid form at room temperature and becomes gel with increasing temperature. Chitosan-*β*-glycerophosphate hydrogels have been researched and used in many studies. This study investigates the various factors that influence the gelation mechanism, such as gel formation rates, temperature, pH, time, and gel specificity. Hydrogels are used in many drug delivery systems and diseases, including nasal drug delivery, vaginal drug delivery, wound healing, peritoneal adhesion, ophthalmic drug delivery, tissue engineering, and peptide and protein delivery. Overall, the chitosan-*β*-glycerophosphate hydrogel is a suitable drug carrier for a wide range of drugs. It shows little toxicity to the body, is biodegradable, and is compatible with other organs. This system can be used in different conditions and different medication ways, such as oral, nasal, and injection.

## 1. Introduction

Today, with the advances in technology and science, new pharmaceutical formulations have been investigated. One of the new types of systems is smart hydrogels, which become gel based on environmental factors or specific conditions, stay in place for a certain period, and slowly release the drug. In these systems, a certain amount of the drug is delivered to the target site, and other tissues are less damaged. Hydrogels are of various types, including temperature-sensitive, pH-sensitive, ion change-sensitive, and magnetic field-sensitive. In this study, a variety of temperature-sensitive hydrogel, chitosan-*β*-glycerophosphate, was investigated and evaluated. It has a liquid form at room temperature but becomes gel with an increase in temperature. This hydrogel can deliver a wide range of drugs. In this study, we investigate the various factors that influence the gelation mechanism [1, 2].

Hydrogels are three-dimensional hydrophilic polymers that can absorb large amounts of water [2] and are used for therapeutic applications since 1960. Hydrogels can provide networks physically or chemically. This network density controls the porous structure of the hydrogels. Hydrogels can be used for drug delivery and tissue engineering purposes because of their biocompatibility, high water content, low surface tension, and mechanical properties similar to those of hydrogels with body tissues. Physically networked hydrogels are soluble in water, which becomes gel due to changing environmental conditions such as temperature, pH, light, electric field, and ionic strength. In fact, there is no covalent bond between the polymer chains of these hydrogels. In contrast, there is a covalent bond between the polymer chains of chemically networked hydrogels. Natural polymers, often called biopolymers, have long been used to develop hydrogels [3]. The highly porous structure hydrogels can be adjusted by controlling the density of cross-links in the gel and the amount of hydrogel bonding in the aqueous medium in which they are located. The hydrogel structure's porosity allows the drug to be loaded into the hydrogel and then released in a rate-dependent manner on the penetration coefficient of small or large molecules into the gel network [4].

Hydrogels have hydrophilic and hydrophobic portions that prevent the polymer from dissolving in water [5]. Hydrogels can entrap water molecules (≥90% *w*/*w*) into their structure through their hydrophilic groups, such as the amine (NH_2_), the carboxylic acid (COOH), and sulfate (SO_3_H) [6]. Hydrogels can be swollen reversibly, and their swelling properties largely depend on their surroundings factors [7–9]. The temperature influences interactions between the hydrophilic and hydrophobic units with the water molecule. Therefore, these changes can increase the solubility of the cross-linked network. This factor is the leading cause of the solid phase change from liquid to solid [10]. The change in the equilibrium between hydrophilicity and hydrophobicity determines the cross-links' dissolution behaviour [11].

## 2. Stimuli-Sensitive Hydrogels

Smart hydrogels are used to release the drug in the body and deliver the protein drugs. The rate of drug release from this system can be modified with changing environmental conditions. Smart hydrogels can be controlled by different changes in their swelling behaviour and mechanical strength in response to various stimuli such as pH, temperature, light, and electric or magnetic field. They are swollen or wrinkled with minor changes in environmental conditions. The water molecules interact with the hydrogel hydrophilic groups in the aquatic environment and form a regular arrangement around the hydrogel strands. When there is hydrogel swelling, it can be fragmented and dissolved if the network connections are degradable [12].

### 2.1. Temperature-Sensitive Hydrogels

Temperature-sensitive hydrogels are liquefied at room temperature but become gel at a physiological temperature or above that temperature. Chenit and Cho [13] showed that the mechanism of chitosan-glycerol phosphate hydrogel gelation depends on several factors, including the following:

1. Hydrogen bonds between chitosan chains—electrostatic interactions and hydrophobic interactions have the main effect on gel formation
2. The addition of chitosan increases the ionic strength, which reduces electrostatic repulsion between chitosan chains
3. Electrostatic attraction occurs between the ammonium group's opposing forces in chitosan and the phosphate group in glycerolphosphate

Moreover, hydrophobic groups in glycerolphosphate improve hydrophobic interactions between chitosan molecules [13].

#### 2.1.1. Classification of Temperature-Sensitive Hydrogels

Among the hydrogels, chitosan-based hydrogels are mostly considered for applications due to their biocompatibility and biodegradability, delivery of hydrophilic and hydrophobic drugs, relative pH stability, and easy formulation (Table 1) [26]. In this regard, several studies reported the applications. Bhattarai et al. [27] loaded PEG into chitosan to form a reversible hydrogel without binding. They developed hydrogels based on poly ethylene glycol-graft-chitosan (PEG-g-chitosan) without additional binders. Poly(ethylene glycol)-graft-chitosan was obtained by binding monohydroxy polyethene glycol to a chitosan scaffold.

By optimising the content and molecular weight of PEG in PEG-g-chitosan polymer, a temperature-dependent reversible phase transition is achieved at the normal room temperature (22–25°C), which is converted to gel at the body temperature (37°C). The phase change stage is directly related to the chitosan chains and the decrease in the polyethene glycol junctions' mobility at high temperatures. In one study, the release of bovine serum albumin from PEG-chitosan hydrogels was assessed. It was observed after a sudden release of the drug at the beginning of the administration, and the residual amount of the drug was released within 70 hours [28]. Chitosan properties can be modified by incorporating hydroxy butyl groups in its main chain to improve its solubility, temperature sensitivity, and plasticity, and the gel is formed at body temperature [29]. The mixture of chitosan and gelatine can produce hydrogels for topical drug delivery and slow release of protein and peptide [30].

A new temperature-sensitive chitosan hydrogel system has been developed from a combination of chitosan and poly (vinyl alcohol) (PVA) used for protein transfer. This hydrogel is formed by hydrogen bonds between the chitosan and PVA chains, and the hydrophobic interactions of the chitosan chains [31]. Adding hydroxyapatite to the chitosan-PVA hydrogel creates a composite that has high tensile strength. The hydrogel of chitosan and potassium hydrogen orthophosphate dipotassium hydrogen (orthophosphate (chitosan/DHO)) can be considered very effective as a controlled-release system in orthopaedic diseases [32]. For instance, doxorubicin (Dox)-loaded chitosan/DHO hydrogel was used clinically to evaluate its therapeutic effect on the orthopaedic osteosarcoma (OS) model. Significant reductions were observed in both primary and secondary osteosarcoma models, and the hydrogel system also reduced Dox-induced cardiac and skin toxicity in mice. Moreover, they evaluated the effect of Pigment epithelium-derived factor delivery with hydrogel on the inhibition of osteosarcoma. The results showed that a combination of plasmid treatment and chemotherapy, combined with the use of chitosan/DHO hydrogels, resulted in significant inhibition of tumour cell growth without adverse effects [32].

#### 2.1.2. Positive and Negative Temperature-Sensitive Hydrogels

Positive temperature-sensitive hydrogels' solubility in water is increased by increasing temperature. Inversely, negative temperature-sensitive hydrogels are shrunk by increasing temperature. At low temperatures, hydrogen bonds are formed between hydrophilic groups of polymer chains and water molecules. As the temperature increases, the hydrophobic interactions between the hydrophobic groups become more vigorous, while the hydrogen bonds become weaker [33]. Changing the equilibrium between hydrophilicity and hydrophobicity determines the dissolution behaviour of the cross-links [11]. The hydrogel is soluble below the Lower Critical Solution Temperature (LCST), while when exposed to the high LCST, it becomes hydrophobic and insoluble, and the gel is formed. The LCST can be modified by changing the hydrophilicity/hydrophobicity ratio. Another hydrogels group is liquefied at temperatures above the Upper Critical Solution Temperature (UCST) and gel at temperatures below this temperature [34].

### 2.2. Chitosan

Chitosan is a biopolymer that has been extensively studied recently [35]. It is a linear polysaccharide formed by the deacetylation of chitin. Chitin is one of the significant components of the outer skeleton of crabs, insects, and the fungal wall [27]. Chitosan is composed of D-glucosamine and n-acetyl-D-glucosamine units, which are randomly interconnected. Chitosan is approved by the US Food and Drug Administration (FDA) [36] for its nontoxicity, biodegradability, biocompatibility, and bioavailability, and has been widely used in engineering and medical fields [26]. Indeed, its unique properties, such as biodegradability, nontoxicity, antibacterial effects, biocompatibility, and adhesion mucus, have led to its wide use as a biomaterial for tissue engineering applications and drug delivery [37, 38]. The results of various studies have shown that chitosan had appropriate wound-healing effects and reduced inflammation. Also, biological properties such as bio-adhesion, anti-cancer, antimicrobial, inflammatory and nociceptive, antioxidant, blood coagulant, and cholesterol-lowering agents make it distinct from other biopolymers [39, 40]. It has been used for over a decade as a safe compound in drug formulation and also, because of its adhesion properties, can be used as an effective material in bonding hard and soft tissues [34, 41]. Chitosan films with a low degree of deacetylation are very suitable for wound healing. They attach to the surface of the tissue and increase the number of keratinocytes, thereby producing new tissue [42]. Chitosan also has an antiacid activity, which prevents the reduction of the effect of drugs on the stomach [43]. Chitosan-based polymer systems are also used to deliver and release proteins/peptides, growth factors, painkillers, antibiotics, antibiotics, cancer drugs, and inflammation, and to treat gene deficiencies. [44]. Chitosan solubility in weak acids is the best justification for its popularity with chitin due to the presence of the first type of amine groups. Its biodegradability by several enzymes and the formation of gels at low pH has made it a unique biopolymer [45].

One of the most recent chitosan applications in the design of slow-release drug delivery systems is the use of chitosan-glycerol phosphate temperature-sensitive solution as a biodegradable gel-forming system at the injection site. Ohya et al. [46] first reported on the use of chitosan nanoparticles in drug applications. They prepared water in oil emulsion of chitosan and then added glutaraldehyde into the emulsion for forming cross-links between the citizen amine groups. Finally, the nanoparticles were created, and 5-fluorouracil, an anticancer drug, was loaded on them. Due to the 5-FU derivatives having an amine-terminal group in their formulation, glutaraldehyde's addition forms similar linkages to the chitosan chain between them. As a result, the drug binds to the polymer chain and becomes completely immobilised. One study demonstrated that it is possible to synthesise stable chitosan nanoparticles capable of holding and delivering the drug [46].

As mentioned, chitosan is soluble in water and has a cationic nature. This feature enables chitosan to interact with polymers or macromolecules that are anionic in nature and have certain polyanions in the aqua environment. These interactions are used to make chitosan nanoparticles. The ability to form gels in contact with anionic groups is an interesting feature of chitosan called ionic gelation (Ionotropic Gelation). This gel formation process is due to the formation of intrachain and interchain cross-links caused by anionic groups' presence. The most well-known reaction is the chitosan reaction with triphosphate groups (TPP) [47]. Anionic TPP as the cross-linking agent for the preparation of chitosan nanohydrogels is one of the most well-known methods for preparing chitosan nanohydrogels. In one study, insulin-loaded chitosan nanoparticles were prepared by mixing insulin with the TPP solution and then slowly adding this compound to the chitosan solution while stirring. By this method, the insulin loading rate was optimised and reached 55% efficiency [48]. Many studies proved that the bioavailability of peptides and proteins is improved by this method for oral delivery. The study also claimed that the bio-stickiness of chitosan enhances the adhesion of drugs to the gut. Zhou et al. [49] studied different formulations of chitosan-TPP nanoparticles prepared by the ion gelation method. TEM images showed that the shape of the particles was spherical, and the diameter of the resulting particles was 20 to 200 nm [50]. Chitosan has a remarkable ability to adhere to mucosal surfaces in the body. In fact, this feature has drawn attention to this polymer in mucosal drug delivery. This unique ability of chitosan has been used to open tight junctions between mucosal cells [51, 52].

### 2.3. Temperature-Sensitive Chitosan Hydrogels

Chitosan is dissolved in dilute organic acid solutions but is insoluble in high concentrations of hydrogen ions at pH 6.5 and is precipitated as a gel-like compound. Chitosan is positively charged by amine groups, making it suitable for binding to negatively charged molecules. However, it has disadvantages such as low mechanical strength and low-temperature response rate; it must be combined with other gelling agents to improve its properties [38, 53]. Using glycerolphosphate salts (possessing a single anionic head) without chemical modification or cross-linking, the pH-dependent gelation properties can be converted to temperature-sensitive gelation properties [54]. In the year 2000, Chenite was the first to design the temperature-sensitive chitosan hydrogels drug delivery system using chitosan and *β*-glycerol phosphate [17]. This new system can remain in the liquid state at room temperature, while becoming gel with increasing temperature above the physiological temperature (37°C). Phosphate salts cause a particular behaviour in chitosan solutions, thereby allowing these solutions to remain soluble in the physiological pH range (pH   7), and they will be gel only at body temperature. When the liquid solution of chitosan-glycerol phosphate, containing the drug, enters the body through a syringe injection, it becomes a water-insoluble gel at 37°C. The entrapped drug particles between the hydrogel chains will be gradually released [38, 53].

### 2.4. *β*-Glycerophosphate

Beta (*β*)-glycerol phosphate is an organic compound found naturally in the body. It is commonly used as a phosphate source in treating phosphate metabolism imbalances and has been approved by the FDA for intravenous administration. *β*-glycerophosphate is considered an osteogenic factor in the culture medium of human bone marrow stem cells, which leads to the differentiation of bone marrow cells to bone cells [55]. It is also used as a serine-threonine phosphatase inhibitor. This compound is often used together with other inhibitors to inhibit a wide range of phosphatases and proteases. Moreover, *β*-glycerophosphate is used as a catalyst in chitosan temperature-sensitive hydrogels and converts fluid to gel phase in chitosan solutions at pH and physiological temperatures. Chitosan hydrogels have been prepared in various formulations, including liquid gels, granules, films, tablets, capsules, microspheres, microparticles, and nanofibers [47, 56]. In each preparation, polymer bonding is performed using noncovalent auxiliary bonds, such as hydrogen, ionic, hydrophobic and physical bonds, or by covalent chemical cross-linking [41]. Related physical joints can often be obtained by mixing the constituents of the gel under appropriate conditions. Besides, gelation occurs without the need for a toxic binding molecule to the body. In this way, it will have healthy clinical applications forever [49]. The *α*/*β*-glycerophosphate is a mixture of *α*-glycerophosphate and *β*-glycerophosphate. *α*-glycerophosphate has a linear chain structure that easily intercalates between chitosan molecules, whereas the hydrophobic force between chitosan molecules is more easily formed. This hydrophobic force causes the water molecules to lie in the chitosan chains' chimney and develop a regular arrangement around them, resulting in the chitosan being dissolved in water. Therefore, *α*/*β*-glycerophosphate may have a similar gelation capacity compared with *β*-glycerophosphate [57]. In this regard, Wu et al. showed that by combining chitosan and *α*/*β*-glycerophosphate and chitosan and *β*-glycerophosphate, temperature-sensitive hydrogels could be prepared [57].

### 2.5. The Mechanism of Chitosan-*β*-Glycerophosphate Hydrogel and Factors Influencing Gelation

**Figure 1:**
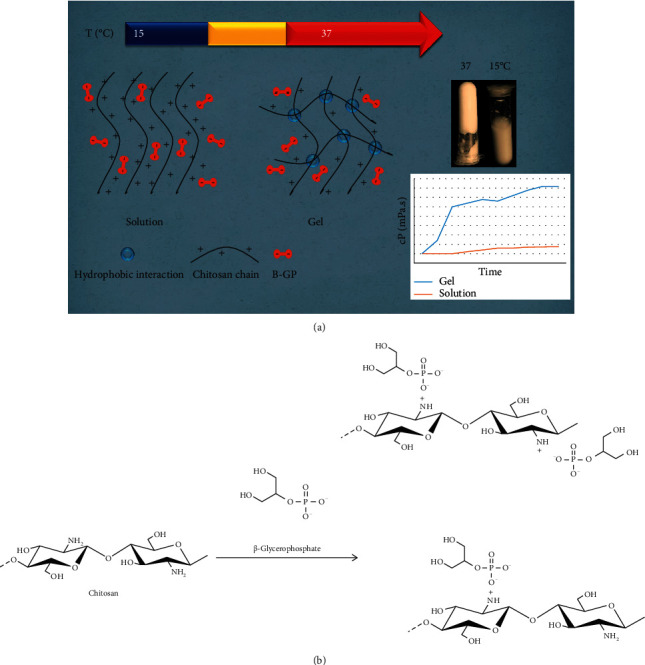
In an acidic environment, chitosan is positively charged (a). Because of the similar groups on the chitosan chain in an acidic environment, the two chains move away from one another, forming an electrostatic repulsion force between the chitosan chains, the water molecules being positioned between the chitosan chains and forming a regular arrangement (b). Adding beta-glycerophosphate, the negative charge of the positively charged chitosan phosphate group forms weak bonds. These weak interactions enhance the water arrangement around the chitosan chains. As a result, chitosan dissolves at physiological pH. Nevertheless, with increasing temperature, the regular arrangement of water molecules around the chitosan chains becomes unstable, the polymer-polymer interactions overwhelm the solvent-polymer interactions, and the chitosan precipitates at high temperature in the form of gels [54].

The polyols surround the chitosan and provide a protective and hydro-resistant layer around the chitosan chains through weak intermolecular interactions such as hydrogen bonding. Increasing the temperature eliminates this polyol layer and allows the polymer to be in equilibrium through stronger hydrophobic bonds, thereby generating gels (Figure 1) [1]. The molecular mechanism of gelation may be due to multiple interactions between chitosan, *β*-glycerophosphate, and water [58]. Effective interactions responsible for converting liquid to gel are: (1) An essential role of *β*-glycerophosphate salts as a reducing electrostatic repulsion agent is to increase the hydrogen bonding between the chitosan chains; (2) Electrostatic attractions of chitosan- *β*-glycerophosphate through ammonium groups and phosphate groups; (3) Chitosan-chitosan hydrophobic interactions are enhanced by the structural role of *β*-glycerolphosphate on water, and the concentration of chitosan and *β*-glycerophosphate influences the soluble behaviour of chitosan and *β*-glycerophosphate hydrogels.

Due to the phosphate groups' neutralising effect, increasing *β*-glycerophosphate concentration slightly increases the chitosan solution's pH. Increasing pH and increasing polymer concentration consume H^+^ ions in the solution through the amine groups. The gelation temperature is gradually decreased with increasing *β*-glycerophosphate concentration and chitosan concentration. However, a synergistic effect at high *β*-glycerophosphate and high chitosan concentrations result in a sudden drop in gelation temperature and phase transition due to the gels' concentration and heat boundary. Increasing the temperature does not affect the pH of chitosan-glycerophosphate hydrogel system and its conductivity. The results show that the ionic interactions are decreased at high temperatures by decreasing the ratio of NH3 ion groups to chitosan and phosphate group to beta glycerolphosphate [59]. In fact, *β*-glycerophosphate plays three major roles in the system: (1) Increases the pH in the physiological range (from 7 to 7.4); (2) Prevents sudden deposition or rapid gelation; (3) Creates some control over the formation of the gel by increasing the temperature.

The temperature of liquid-to-gel conversion is sensitive to the environment's pH and the gelation time is temperature-dependent. Depending on the material's gelation behaviour, the hydrogels' rheological properties follow three types: (1) A liquid-like behaviour at low temperature; (2) A fast gelation process at a temperature near the gelation point; (3) A slow gelation process at high temperature. When the temperature is increased, the water molecules are exited from the chitosan chains, and the heat accelerates the proton transfer from the protonated amino acids to glycerophosphate. As a result, the formation of hydrophobic interactions between Chitosan chains is facilitated. In the study of rheological behaviour, the system shows the lowest level of contact obtained by the Ostwald ripening process. Also, the molecular weight and the degree of citizen distillation affect the chelation process [54, 59]. It has been observed that the gelating point is 33°C for the lower molecular weight chitosan sample, which is 2°C higher than the high molecular weight chitosan sample. It means that hydrophobic interactions occur in lower molecular weight chitosan samples at higher temperatures. It can be the result of more cross-links and shorter hydrophobic groups. Such an observation can be attributed to the low molecular weight and low viscosity of the sample, thereby increasing the possibility of accumulation and penetration of hydrophobic groups. The lower molecular weight facilitates the accumulation and penetration of hydrophobic groups. Therefore, gelation occurs at a lower temperature.

The deacetylation of chitin chains to get more chitosan occurs at amorphous sites. Thus, the type of hydrophobic and hydrophilic joints was the main result of this deacetylation. Therefore, deacetylation affects the gelation process by simulating the hydrophobic properties of chitosan. Chitosan can be considered a bond of two polymers consisting of deacetylated and acetylated units. Moreover, this gelation can occur depending on the degree of chitosan deacetylation at lower temperatures [60]. In this way, the higher degree of chitosan deacetylation results in an acceleration in the gelation speed. It may be due to the increased bond density and cross-linkages between the phosphate groups of the glycerophosphate molecule and the ammonium groups of chitosan.

The gelation time is decreased with increasing *β*-glycerophosphate content, and gelation occurs at lower temperatures and at a lower time. By increasing the chitosan concentration, the gelation rate is increased, but the gelation occurs at a higher temperature (2% *w*/*v* is the best concentration). However, by reducing the molecular weight of chitosan, the gelation time is increased. On the other hand, gelation temperature can be decreased and varied in the range of 37–32°C by increasing chitosan deacetylation degree, increasing *β*-glycerophosphate concentration, increasing pH (6.8–6.8), or reducing chitosan molecular weight [61, 62].

Preparing the temperature-sensitive hydrogels is dependent on the hydrophobic-hydrophilic reactions of the chain molecules. The results show that the gelation time, structure, swelling rate, and degradation rate in the in vivo and in vitro environments depend on the cross-links and the hydrogel structure. It has been observed that the gelation and swelling times depend on the binding, overlap, and adsorption of the polymer chains at different pH and temperatures. Finally, it can be said that in aqueous solutions of chitosan-glycerol phosphate, (1) the electrostatic attractor amine and phosphate groups stabilise the solubility at ambient temperature and (2) hydrogen bonds between the chains along with the chitosan-chitosan hydrophobic interactions cause the gel formation at body temperature (Figure 1) [63].  Table 2 shows several factors affecting gelation time.

### 2.6. Chitosan-Glycerophosphate Hydrogel Derivatives

**Figure 2:**
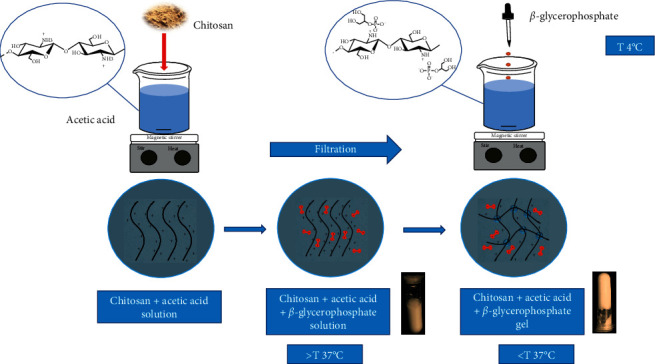
The schematic figure for preparing the chitosan-based hydrogel.

Chitosan and its derivatives become thermally sensitive by adding glycerophosphate to a 2% *w*/*v* chitosan solution (Figure 2) [69, 70]. The rate of degradation of chitosan-based hydrogels largely depends on the cross-linking of another class of gel-forming compounds such as gelatine and genipine [71, 72]. The drug release profile of chitosan hydrogels is affected by the degree of hydrophobicity of the drug. Compounds such as latanoprost and ferulic acid, which have low solubility in water (≤1 mg/mL), are slowly released from hydrogels (28% in the first 24 hours). But, water-soluble compounds such as timolol maleate (2.74 mg/mL) have a high release rate (almost 80% of the drug in the first 24 hours). Chitosan hydrogels with 100% gelatine or cross-linked with a secondary linker, such as genipine or hydroxyapatite, will not have a 100% release in the long run. These secondary cross-links cause slower drug release due to the creation of smaller pores within the hydrogel networks [70, 73].

Chitosan-*β*-glycerophosphate hydrogels have no sufficient mechanical properties for some drug delivery systems, including for cell loading. Due to its biocompatibility properties, degradability, and ability to support cell growth, collagen and its derivatives, including gelatine, were selected as the first polymer to modify the properties of hydrogels. In one study, the addition of 2% gelatine to chitosan/glycerophosphate solution reduced the gelation temperature from 36°C to 31°C and reduced the gelation time at 37°C from 10 minutes to 2.3 seconds [1]. The mechanical strength of hydrogels is also improved with increasing gelatine concentration. Furthermore, chitosan/gelatine/glycerophosphate hydrogels have all of the biocompatibility and biodegradability properties found in chitosan/glycerophosphate hydrogels. Nevertheless, the addition of gelatine reduces the solubility of the solution at room temperature. Even at room temperature, the gel formulation can be done, but this can be reduced by lowering the gelatine concentration [74]. Sodium hydrogen carbonate (NaHCO_3_) is applied to produce high-strength chitosan-*β*-glycerophosphate hydrogel. Hydrogels that contain large amounts of NaHCO_3_ have shorter gelation time and are more resistant in compression. These hydrogels create a highly porous structure, which provides suitable conditions for cell growth and has good biocompatibility at low *β*-glycerophosphate concentrations. Therefore, adding appropriate amounts of NaHCO_3_ to the chitosan *β*-glycerophosphate hydrogel can improve its properties [75]. Wu and his colleagues developed a temperature- and pH-sensitive hydrogel from the combination of chitosan and quaternized chitosan (HTCC) containing *α*,*β*-GP (CS-HTCC/GP), which investigated doxorubicin's release behaviour in various modes [57, 76]. Chitosan-HTICC-GP hydrogels are also used for nasal drug delivery. Placing insulin on the hydrogel and using it through the nose reduced blood sugar by 50–50% for 4–5 hours. In addition, this hydrogel is highly biocompatible and easily degraded and excreted by the body [38].

## 3. Advantages of Temperature-Dependent Chitosan-*β*-Glycerophosphate Hydrogels

### 3.1. Biocompatibility and Biodegradability

One of the major advantages of chitosan hydrogel systems, unlike other drug delivery systems such as implants, is the degradable system that eliminates after a while and does not require surgery to remove the carrier. Chitosan is attacked and destroyed by the body's hydrolytic enzymes such as lysozyme [27, 77]. Lysozyme in various fluids and tissues in the human body are found, with concentrations ranging from 4 to 13 mg/l. The results show that the rate of degradation of chitosan hydrogels is correlated with the degree of chitosan deacetylation. If the chitosan deacetylation degree is high, the rate of hydrogel degradation is lower [78]. By increasing the concentration of chitosan, the rate of degradation is decreased. Hydrogel degradation by lysozyme begins at its surface and gradually penetrates it. Continuous degradation of hydrogels occurs through the hydrolysis of acetyl groups. The hydrogel network's porous structure is maintained for seven days *in vitro* while significantly reduced and degraded *in vivo*. Similar studies have shown that after intramuscular injection of the hydrogel, it begins to degrade after eight weeks and is completely degraded after 12 weeks [79]. The destruction of hydrogels in the body should be considered on the drug pharmacokinetics because it may alter the rate of drug release in the long term [1].

### 3.2. Porous Structure and Heterogeneous Morphology

Ahmadi and Borujen revealed that chitosan-glycerolphosphate hydrogel could stimulate mesenchymal stem cell (MSC) proliferation at specific concentrations, indicating that it would be a suitable carrier for cell encapsulation and tissue engineering [80]. The morphology of chitosan glycerolphosphate was examined by laser scanning confocal microscopy (LSCM), and the results showed that the hydrogel had a completely heterogeneous morphology [81].

### 3.3. Controlled Release and Sustained Release

Kempe et al. found that insulin in the aqueous medium had its original spin-labeled hydrogel and was naturally released from the formulation [10]. Besides, they claimed that insulin is released into the chitosan hydrogel matrix by diffusion and that the released drug increases with increasing glycerolphosphate ratio. Observations show that the drug release is slower when added to the chitosan solution alone than when the drug is added directly to the chitosan solution combined with glycerol-phosphate, in which chitosan-glycerol phosphate hydrogel can take the drug for several days. It has also been observed that the release of hydrophobic drugs such as Adriamycin® occurs less rapidly than hydrophilic drugs such as 6-mercaptopurin from chitosan glycerolphosphate hydrogel [82].

### 3.4. Drug Targeting

Hydrophobic drugs are first inserted into the liposome and then transferred to chitosan glycerol hydrogels to achieve a slow release. It was observed that the administration of *in situ* chitosan glycerolphosphate hydrogel containing paclitaxel prevented the recurrence of the tumour [83]. Chitosan glycerolphosphate hydrogel has also been used to transport cells into cartilage tissue for its repair [65]. In a study, ellagic acid was firstly loaded into PLGA and subsequently embedded in chitosan-glycerolphosphate hydrogel to improve its bioavailability and slow its excretion. As result, it was found that ellagic acid had a slower release and more appropriate bioavailability [84]. Moreover, Han et al. reported that hydrogel chitosan glycerolphosphate combined with chemotherapy drugs and viral vaccines had synergistic antitumour effects against the tumour [85]. *In vitro* studies of hydrogel chitosan glycerolphosphate containing Ellagic acid for brain cancer have been studied [26]. Recently, the use of hydrogel polymers similar to chitosan glycerolphosphate and elastin in bone tissue engineering has expanded and improved the mechanical properties of tissue under physiological conditions [8].

## 4. Method for Preparation of Temperature-Sensitive Hydrogel Chitosan-Glycerophosphate

The hydrogel can absorb much water as an effective drug delivery system that creates a porous structure [27, 86]. Chitosan powder with a deacetylation degree more significant than 85% and a molecular weight of more than 650 kD is commonly used to prepare hydrogels (Figure 2). The method is described as follows [66]. First, we dissolve a certain amount of chitosan in a 0.1 M acetic acid solution and vigorously stir for 1 h. We must remove the precipitated chitosan by filtration, then cool and place it at 4°C for storage. The prescribed amount of active ingredient was dispersed in 1 mL distilled water and treated by ultrasonication for 1 h. Dissolve 300 mg of glycerolphosphate in 1 mL of distilled water and pass the resulting solution through a 0.22 *μ*m filter. In the second step, 1 mL of active ingredient was added into the chitosan solution and stirred for 1 h until well dispersion. If the hydrogel must be sterile, then autoclave all solutions separately at 121°C for 15 minutes, cool to room temperature and store at 4°C, and perform the rest of the steps in a sterile place (Like a laminar flow hood). Finally, the glycerophosphate solution was added dropwise to the active ingredient/chitosan mixed solution under constant stirring at low temperature. As the control group, the active ingredient/chitosan hydrogel was prepared following the same steps above, except that the active ingredient dispersion was replaced by 1 mL distilled water. Store it in the fridge. To investigate the morphology of the hydrogel, we use an SEM microscope. Put the hydrogel formulation at 37°C for 3 hours, then freeze it. Place the lyophilised powder at the desired location and coat the surface with a thin layer of gold [66].

## 5. Sterilisation

Injectable medications must be sterile and free of pathogens before usage. The final sterilisation process requires high temperature, such as steam or high heat, which cannot be used because of the hydrogel's thermosensitive property and the irreversibility of the chitosan-glycerophosphate hydrogels is impaired [87]. Therefore, gamma irradiation is one way of ultimate sterilisation, which reduces the hydrogel formulation's viscosity even at temperatures below 80°C due to the polymer chains' degradation [88]. On the other hand, sterilising the product using 0.2-*μ*m filters can be complicated depending on the viscosity of the solution or may not be possible if the drug or cells are dispersed in the solution [1]. In general, chitosan and *β*-glycerophosphate solutions are sterilised separately and mixed in aseptic conditions. Sterilising chitosan solution with steam before adding glycerophosphate is one of the common ways. However, this method directly damages the polymer and reduces its porosity and affects its viscosity [89]. Chitosan powder or solutions can be sterilised by ultraviolet, gamma-ray, or beta radiation, but these methods may damage the polymer structure and usually are not considered suitable. The best way to sterilise chitosan powder using water vapour seems to maintain the chitosan's molecular weight and then dissolve the chitosan in the acidic solvent under sterile conditions [90].

## 6. Applications of Temperature-Sensitive Chitosan-Glycerophosphate Hydrogels

**Figure 3:**
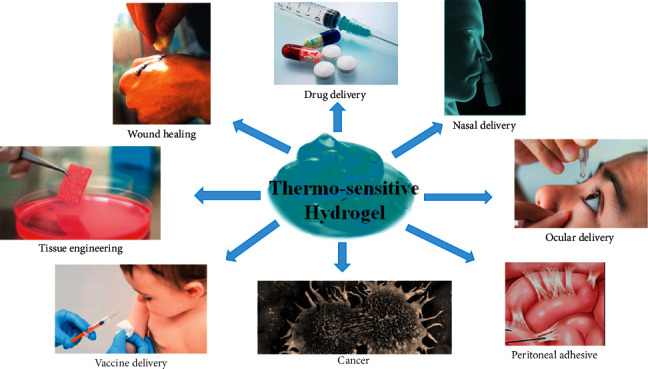
Applications of thermosensitive hydrogel.

Hydrogels are widely used to control and slow down the release and prolong drug delivery. Nowadays, It has several applications in tissue engineering and wound repair (Table 2 and Figure 3) [82]. Subcutaneous or intramuscular injection of the formulation can increase the release time from one day to one month, depending on the concentrations of chitosan and glycerophosphate, and the properties of the loaded drug. Several drugs have been studied with this method, such as aspirin [62], risperidone [91], insulin [53], methylprednisolone [92], docetaxel [67], venlafaxine [66, 93], doxorubicin [66, 94, 95], curcumin [66], and some antibiotics, such as mitomycin [65], as carriers for tissue engineering and cell encapsulation [96] and protein and peptide [97], such as erythropoietin [64, 98] and nerve growth factor [71].

### 6.1. Drug Delivery System

#### 6.1.1. Nasal Delivery

Temperature-sensitive hydrogels are an excellent option for nasal drug delivery. They are liquid at room temperature, easily dispersed in the nasal cavity during use, and then transformed into a gel at body temperature, remaining on-site for a long time, improving bioavailability and drug absorption [99, 100]. Chitosan is a biocompatible, biodegradable, and nontoxic polymer widely used as an effective carrier for enhancing drug absorption through the nasal passage. Chitosan binds weakly to the sialic acid present in the nasal mucosa, thereby creating a gap between the tight epithelial junctions and opening the drug delivery pathway, and increases the absorption of drugs. Moreover, chitosan also acts as a mucosal adhesive compound, increasing drug retention duration on the mucosa, increasing its bioavailability and uptake [101, 102]. Further compounds, such as polyethene glycol and hydroxypropyl methylcellulose, are added to the formulation to improve the temperature-sensitive and adhesive properties of chitosan hydrogels [76]. However, these auxiliary interactions can irritate the nasal mucosa [103]. On the other hand, drug and carrier interactions lead to drug crystallisation inhibition and improve drug solubility [104]. Molecular interactions, especially hydrogen bonds between polymer chains and drug molecules, play a major role in drug efficacy [103].

Moreover, thermosensitive hydrogel formulations of chitosan are used to transport biological substances such as genes and proteins. However, there are limiting factors as well; for example, chitosan is soluble and can release hydrogen only in an acidic environment [105]. Substitution of trimethyl chitosan chloride derivatives instead of chitosan in chitosan-*β*-glycerophosphate hydrogels retain the primary polymer's critical properties but enhance its solubility, adhesiveness, and temperature-sensitive properties, and activate the product at a broader range of pH [57, 106]. A nasal formulation should be formulated to be liquid at room temperature and also used via spray. It becomes a gel at 32–35°C from the liquid state and has good rheological and adhesive properties for remaining in the nasal mucosa for a long time. In one study, hydrogel formulations were prepared from the combination of polyethene glycol and glycerophosphate with n-tri-methyl chitosan, which was gelled at 32.5°C for 7 minutes. This hydrogel provided good rheological properties to the mucosa and was well placed on the mucosal surfaces. It seems that this formulation could be a good choice for nasal drug delivery [107].

Temperature-sensitive hydrogels are an ideal system for delivering vaccines through the nose. These systems are used to transport fragmented H5N1 and Ebola antigens. This system disperses the vaccine well into the nasal mucosa, holds the antigen on the nasal mucosa for a long time, and opens tight junctions of the nasal epithelial cells, thereby increasing vaccine uptake. Therefore, chitosan-*β*-glycerophosphate hydrogels can also be used for vaccine delivery [108].

#### 6.1.2. Ocular Delivery

In a study in 2015, the effect of chitosan hydrogel containing ferulic acid on burn injury in the cornea was assessed. Topical use of chitosan-ferulic acid hydrogel drops improved the rate of corneal healing compared to the control group. It was also observed less hyperplasia on the corneal surface in the group treated with a Hyperplasia was also lower on the corneal surface in hydrogel containing ferulic acid group (pH = 7.4) [76]. Moreover, one study in 2016 represented that chitosan-gelatine-glycerolphosphate hydrogels containing latanoprost could significantly reduce the intraocular pressure following the triamcinolone acetone-induced glaucoma in the rabbit model. In this study, a temperature-sensitive hydrogel was designed wherein gel formation occurred at 34.18°C and provided a stable release profile. Due to its adhesive mucosal properties, chitosan has long been attached to the eye's mucosa and provides a longer release profile. After one week, the intraocular pressure was significantly reduced compared to the control group (one drop per week). Finally, it was observed that the pressure in the eyes was within a normal range. This formulation was converted to gelling at 34.18°C, initially assumed to be below the eye temperature, but it was observed that the formulation became gel in approximately 70 seconds. In this study, the deacetylation rate of chitosan was more than 95% [109].

#### 6.1.3. Lung Delivery

Chitosan-*β*-glycerophosphate hydrogels have been studied in the treatment of pulmonary emphysema. In emphysema, the elasticity and structure of the alveoli are lost, resulting in airway collapse. There is currently no definitive treatment, and it is only possible to slow down its progression and treat its symptoms. In patients with severe emphysema, we have surgery or a lung transplant. Using hydrogels, we can use it as a factor to seal the damaged area. Chitosan hydrogel is used in this case due to its biocompatibility and degradability. We can easily transfer the gel to the target site in the lungs, and by injecting it at the site, it fills the damaged areas. The three-dimensional network structure of the hydrogel and its physical properties, such as stability and adhesiveness, and the high gel rate, provide beneficial characteristics. In one study, chitosan *β*-glycerophosphate hydrogel was used as a lung sealant for dogs with biological lung volume reduction (BLVR). The hydrogel is liquefied at room temperature. It is easily injected into the site of injury and becomes a gel, and remains after a few minutes on the site. After three weeks, it was observed that the chitosan *β*-glycerophosphate hydrogel created low stability and mechanical strength at the site. Thus, a certain percentage of genipine was added as a nontoxic cross-linker, with outstanding results [110].

#### 6.1.4. Vaginal Delivery

Pseudoplastic semisolid formulations are considered more suitable for vaginal administration because they offer increased flow, which helps in extrusion from the applicator and enables avoidance of leakage from the administration site. Chitosan can contact and attach the vaginal surface well due to its sticky mucus properties. One of the challenges we face about chitosan hydrogel is the sudden initial release of the drug. In one study, the drug was placed in a microsphere and loaded into a hydrogel. This procedure significantly reduced the initial and sudden release rate. Mucosal hydrogels improve drug targeting in the vaginal cavity due to prolonged exposure to the drug at the site of infection or inflammation. The parameters that affect the hydrogel properties of vaginal administration are [111] as follows: (1) Cohesiveness: the higher the cohesiveness of the hydrogel, the better because it regenerates faster and forms a gel, improving product performance. (2) Hardness: if the hardness is low, the hydrogel is easily removed from the prescription site, but if it is high, it stays in place longer, and the drug is released for a long time. (3) Inflation: this property depends on the environment's pH, the ions inside the environment, the active groups on the polymer, and the polymer bonds, which affect the speed of drug release. (4) Mucosal adhesive properties: mucosal adhesive properties are the bonds between hydrogel chains and mucosal surfaces. Chitosan *β*-Glycerophosphate hydrogels are adhesive due to the presence of chitosan in the mucosal property [111].

One of the convenient features of chitosan hydrogel is the mucosal adhesive properties and anticandidiasis effects of chitosan. One of the most important features in vaginal delivery systems is that the carriers should be stable in the pH of this area and should not change the physiological pH, which leads to fungal and bacterial infections. Chitosan hydrogel provides a uniform release of the drug into the vagina, but it increases the pH of the site. This uniform release is caused by high water absorption and numerous pores. In general, chitosan *β*-glycerophosphate hydrogels can be a suitable option for vaginal delivery [111].

### 6.2. Diabetes (Insulin)

The chitosan-*β*-glycerophosphate solution provides a constant pH range in the physiological range (6.6–6.8) that is not changed during the gelation. In one study, the insulin release from chitosan- *β*-glycerophosphate hydrogels was investigated. It was observed that insulin is released from hydrogels within two weeks. As the concentration of *β*-glycerophosphate increased, the amount of secreted insulin and its release rate also increased. This formulation contained 2.5% chitosan and 8.16% *β*-glycerophosphate. In this study, it was observed that at least 6% *β*-glycerophosphate must be present in the formulation for carrying out the gelation process. In this study investigating the thermosensitive chitosan hydrogels containing insulin, chitosan-*β*-glycerophosphate hydrogel maintains insulin stability under physiological conditions and can be used as a formulation for the delivery of peptide and protein drugs. They placed different concentrations of insulin in two solutions containing different concentrations of chitosan-glycerophosphate and examined the drug release *in vivo* over three weeks. The results showed that drug release decreased with increasing *β*-glycerophosphate concentration from the formulation. An 8-amino-1-naphthalene sulfonate probe evaluated the stability of the released insulin. The results showed that its natural structure releases insulin. Eventually, the study showed that chitosan hydrogels containing *β-*glycerophosphate could be used to transfer insulin to the body [10, 53].

### 6.3. Cancer

In one study, it has been shown that administration of a thermosensitive chitosan hydrogel containing paclitaxel on EMT-6 murine mammary cancer cells, equivalent to four intravenous injections of this drug, inhibited cell growth. The results show that chitosan-*β*-glycerophosphate hydrogel can control cancer cells' growth even in the absence of the drug. One of these causes may be due to chitosan's antitumour effect and its derivatives inhibiting cancer cells' growth [15]. In one study, chitosan-*β*-glycerophosphate hydrogels containing paclitaxel were prepared. In this study, to increase the solubility of the drug, the drug was first loaded on cyclodextrin, and then to improve the physicochemical properties of the hydrogel, polyvinyl alcohol (PVA) was added to the hydrogel. Studies have shown that Taxol® formulation completely releases the drug during the 24 hours, while hydrogel formulation can release 82% of the drug during the 13 days. It was found that intratumour administration of paclitaxel hydrogels had approximately 3-fold more significant antitumour effects than the commercial formulation of paclitaxel (Taxol®) [112].

### 6.4. Hydatid Cyst

There are several methods for the treatment of hydatid cysts, from surgery to chemotherapy. The most important concern is about injury to the cyst during surgery. If the cyst ruptured during surgery, the material that comes out can cause new cysts to form on the site and cause anaphylactic shock or even death. If we can somehow solidify the fluid inside the cyst, the risk might be reduced. Injectable, temperature-sensitive hydrogels can be a good choice as a gelating agent. In one study, chitosan *β*-glycerophosphate hydrogels were used to control cyst subsidence during aspiration. They swell in aquatic environments and have a high capacity to absorb water or physiological solutions in their network structure. The amount of fluid absorbed in hydrogels depends on the formulation, impurity, and salt content. The reason for choosing this type of gel strength hydrogel is the uniform internal network. The hydrogel solution was injected into the cyst with an insulin syringe, and after a particular time, it became a gel. The gel duration is related to the physicochemical nature of the cyst fluid. The concentration of salt and the type of salt ion can reduce the gelating time. After gelation, the cyst can be removed more easily from its place because the possibility of rupture is reduced [113].

### 6.5. Spinal Cord Injury

Chitosan *β*-glycerophosphate hydrogels can be used as a carrier to transfer cells to the site of injury. In one study, mesenchymal stem cells were loaded into hydrogels. The paracrine function of mesenchymal stem cells improves, repairs, and reduces cell death in the spinal cord. Due to its high biocompatibility and anti-inflammatory and antioxidant effects, chitosan *β*-glycerophosphate hydrogels are an excellent option for mesenchymal stem survival cells. Mesenchymal stem cells secrete a wide range of growth factors, cytokines, and extracellular vesicles, which regulate cell-to-cell and cell-to-extracellular matrix functions. Chitosan *β*-glycerophosphate hydrogels contain large amounts of water and a viscoelastic surface close to living tissue that can simulate the spinal cord's mechanical properties. In this study, it was observed that the mesenchymal stem cells transferred to the damaged spinal cord were able to reduce the volume of the lesion and improve spinal function. However, the study found that the number of mesenchymal stem cells that remained in place after recovery had decreased due to poor environmental conditions at the injury site. Chitosan substrates induced the differentiation of cortical and spinal cord progenitor cells into cortical neurons and motor neurons and promoted active synaptic networks' rearrangement [114].

### 6.6. Wound Healing

Many factors, such as molecular weight, flexibility, cross-linking density, and hydrogen-bonding capacity, affect chitosan hydrogels' adhesive properties. The high molecular weight of chitosan (∼340 kDa) and gelatine can improve the adhesion properties of hydrogen adhesives. Both hydrogel and gelatine molecules have many hydrophilic groups that form hydrogen bonds between them and increase their mucosal adhesive properties and increase the drug release duration [103]. Chitosan is a cationic polymer that can bind to and attach to mucin's negative charge, thus providing excellent mucosal adhesive properties [115]. One of the challenges we are faced with chitosan hydrogels is the sudden initial release of the drug [116]. In a study of chitosan membranes used for wounds healing, it has been observed that the scar formation of burns on the rabbit cornea due to the alkaline burns model is reduced [100].

In another study, vancomycin hydrochloride microparticles were first loaded into the HPMC by the spray dryer method. These particles were found to have an average diameter of 1.5–6.4 *μ*m, had a good dispersion, and made approximately homogeneous particles. The drug was encapsulated at about 72.6%. The drug was then added to the chitosan-*β*-glycerophosphate solution to give a slow-release formulation. The *in vitro* results showed good release kinetics, and the drug was released at a reasonable rate for a long time from the hydrogel [25]. In general, this formulation can release the drug appropriately and prevent wound infection. Studies show that hydrogel is a very suitable formulation for the treatment of chronically infected wounds. However, it has problems such as the high initial release of the drug. In another study, a new formulation of cefuroxime was developed that slowly releases and prolongs the drug's release as a hydrogel matrix made of chitosan. In this study, different concentrations of cefuroxime were loaded in hydrogels. It has been found that phosphate buffer (pH 7.4) released more drugs than the medium containing stearate and alkaline (pH = 10). Studies on the L929 fibroblast and MG-63 osteosarcoma cell lines showed that this hydrogel was biocompatible and could be used for long-term drug delivery in chronic wounds [117].

### 6.7. Cell Therapy/Tissue Engineering

Injectable hydrogels can cause irregular shapes, survive for long in the tissue, and do not stimulate the immune system, so that they can be an appropriate candidate for cell therapy [118]. The hydrogels' porous structure improves drug release into the environment without altering the physical and chemical structure of the drug [119]. The cell encapsulated within the hydrogel can easily exchange materials with the surrounding environment, receive various factors, divide and grow up [120]. Many hydrogels are created using either organic compounds or organic binders that negatively affect cellular activity [96]. Hydrogels can support cells during tissue regeneration and drug delivery systems because the hydrogel's physical and chemical properties are similar to the body's natural extracellular matrix. Tissue engineering (TE) is a promising method for repairing damaged bone tissue. Injectable hydrogels are positioned through a single injection using a wide range of materials used as fillers without intervention and surgery and are used in applications such as bone defect improvement. Chitosan hydrogel has several benefits, including similarity to the extracellular matrix (ECM) and providing a unique microenvironment for cell growth that can be of great use in cell culture [121]. Hydrogels have a high viscosity and high ability to eject the syringe at the time of injection. After ejection from the needle and presence in the environment, their viscosities increased rapidly and became a gel and hardened. The best feature of these hydrogels is their high resistance to internal stresses, making them suitable for cell therapy and tissue engineering.

The following are some of the limitations of cell therapy: ‏(a) the difficulty in producing an adequate combination of gelation kinetics and cytocompatibility, (b) their inferior mechanical properties after gelation, (c) poor survival and weak cell proliferation (d) toxicity of beta-glycerophosphate at a concentration above 0.12 M for cells also cause hyperosmolarity of the hydrogel. To eliminate these limitations, we can add other compounds such as dibasic ammonium hydrogen phosphate, dibasic hydrogen phosphate, or sodium hydrogen carbonate to the hydrogel structure or replace it with *β*-glycerophosphate. Due to the body's physiological pH and the gel's osmolality, cell survival is well preserved. Adequate studies on the presence of pores in the gel and its relationship with cell survival or oxygen diffusion to hydrogel are not available, and further research is required. Studies have shown that chitosan increases the cell viability and distribution capacity of encapsulated fibroblasts in serum and plasma environments. The ability of the hydrogel to absorb the water causes the exchange of nutritious material and ions required by the cell with the surrounding environment. Therefore, it is a suitable environment for the growth of cells and tissues. Hydrogels can create an elastic environment similarly to body tissues. Creating this elasticity and compressive strength by using a biodegradable hydrogel without creating new chemical bonds is an advantage of chitosan. Various studies show that these hydrogels can regenerate soft tissues and even tissues such as cartilage under high pressure [122].

### 6.8. Peritoneal Adhesion

Peritoneal adhesion is considered a significant problem that occurs after peritoneal surgery. In one-third of surgeries, the connective tissue is created between the injured areas, which can cause many problems, including bowel obstruction. Occasionally, after surgeries, such as childbirth, very large adhesions can develop that they can also involve the ovary and cause complete infertility [123, 124]. In a study performed in 2006, chitosan-containing gels were found to have anti-adhesion effects on tissue damage to the peritoneum but did not affect external adhesion factors, such as talc. The chitosan film created in the injured area remains in place for more than two weeks, which can exacerbate its adhesion. The addition of gelatine to chitosan causes the film to be more biodegradable in less time and reduces adhesion probability [104, 105]. In another study, naproxen (a hydrophobic drug) nanoparticles were embedded in chitosan-*β*-glycerophosphate hydrogels. In this study, a rat model was used to induce adhesion and test the formulation. At the end of the study, the hydrogel was easily separated from the peritoneal wall. It has been observed that on the 7th day after surgery, the wounds created during surgery were entirely covered by an epithelial layer; however, lesions were attached and provided severe adhesion in the negative control group. Also, chitosan hydrogel containing naproxen had fewer toxic effects on central tissues and organs, including the liver, spleen, heart, lung, and kidney [106].

## 7. Conclusion

The chitosan-*β*-glycerophosphate hydrogel is an intelligent drug system widely used to deliver hydrophilic and hydrophobic drugs to different parts of the body. It can store a large amount of drug in its three-dimensional network and release it to the environment at a controlled rate at a specific location. Changes in this system's components, such as changes in chitosan concentration, the molecular weight of chitosan, degree of acetylation, glycerophosphate concentration, and the addition of other reagents, can control the formation gel's time, temperature, and pH. Moreover, by increasing glycerophosphate salt concentration, the concentration of chitosan solution, and DDA or ambient temperature, chitosan solutions' gelling time can be reduced. Due to its good features, such as being biodegradable and biocompatible with the human body and the ability to stay in place for a long time and controlled drug release, this drug delivery system can be an excellent option to replace other drug forms of implants. Also, due to its three-dimensional and highly porous structure, this system can provide a suitable environment for the growth and proliferation of a wide range of cells, which will be an appropriate option in the field of tissue engineering. Besides, by adding other compounds, such as gelatine, polyvinyl alcohol, and graphene oxide, to Chitosan-b glycerophosphate hydrogel, changes in the drug release rate and gel formation time can be made. These new systems are more efficient than chitosan beta-glycerophosphate hydrogels. Due to this drug delivery system's desirable features, it is predicted that the drug-containing formulations from this drug system will enter the global market in the future.

## Figures and Tables

**Table 1:** Type of thermo-responsive hydrogels.

	Polymers combined with	Gelling temperature °C	In the market	Disadvantages	Advantages	Application	Reference
*1. Cellulose derivative*							
1.1 methylcellulose	N-isopropyl acrylamide (NiPAAM)	60–80				Create a bioactive scaffold	[14]
1.2 chitosan	PEG [15] N-isopropyl acrylamide (NiPAAM) [16] Glycerophosphate [17]	37	BST-Gel ® [18]	The initial release of the drug is high. It always retains some of the drugs. It has a low rate of destruction	Forms a reversible gel that gels when placed in the body. It can release the drug for a long time. Biodegradable and good biocompatibility	Cell delivery Drug delivery Implant Similar structure to extracellular matrix	[17]
1.3 Dextran	N-isopropyl acrylamide (NiPAAM)	32–37		At a temperature of 37 degrees, its degradation rate decreases. Drug release depends on various factors such as pH and electrolyte	It can create a sustained release formulation	Drug delivery	[19]

*2. Proteins*							
2.1 Gelatine	Poly(ethylene glycol)-Poly (D, L-lactic) (mPEG-DLLA) [20]	Below 25		Improper gelling and adhesion properties The initial release of the drug is high	Slow-release profile	Drug release kinetic with gentamycin sulfate	[21]

*Other polymers*							
3. N-isopropylacrylamide		32		Nonbiodegradable The gelling temperature depends on the pH and electrolytes of the environment. Swelling was lower Initial burst release	Preparation of implants Long-term drug release	Drug delivery Cell encapsulation Cell culture Biomedical engineering application	[22]
4. PEO/PPO Poloxamer ®	Poly (ether-carbonate)	37	LeGoo ® [23]	Lower stiffness than other hydrogels After one week degraded	Biocompatible High viscosity	Drug and gene delivery Tissue adhesive Burn wound covering	[24, 25]

**Table 2:** Factors influencing gelation, chitosan-*β*-glycerophosphate.

MW	Con	DDA %	Time (min)	pH	GP	*T* (°C)	Drug	Reference
310–375	0.8 g	103	5	7	2 g	37	Aspirin	[64]
62	0.1% *w*/*w*	82	1.6	7.4	12%	30	Mitomycin-c	[65]
124	0.1% *w*/*w*	72	1.4	7.4	12%	29.8	Mitomycin-c	[65]
370	0.1% *w*/*w*	71	1	7.4	12%	29.6	Mitomycin-c	[65]
650	20 mg/mL	85	10	7.35	3%	37	Curcumin	[66]
200	1.8% *w*/*w*	91	12	6.88	9 *w*/*w*	37	Docetaxel	[67]
200	1.8% *w*/*w*	91	5	6.97	12 *w*/*w*	37	Docetaxel	[67]
200	1.4% *w*/*w*	91	>120	6.75	6 *w*/*w*	37	Docetaxel	[67]
200	1.4% *w*/*w*	91	61	6.83	9% *w*/*w*	37	Docetaxel	[67]
306.12	2%% *w*/*v*	95	2	7.63	14% *w*/*v*	37	Insulin	[68]
306.12	2%% *w*/*v*	95	3	7.52	12% *w*/*v*	37	Insulin	[68]
306.12	2%% *w*/*v*	95	5	7.45	10% *w*/*v*	37	Insulin	[68]
306.12	2%% *w*/*v*	95	14	6.91	5% *w*/*v*	37	Insulin	[68]

## Data Availability

Data will be available on request to the corresponding author.

## Abbreviations

LCST: Lower critical solution temperature
UCST: Upper critical solution temperature
PEO: Poly (ethylene oxide)
PPO: Polyphenylene oxide (PPO)
PEG: Poly (ethylene glycol)
PVA: Poly (vinyl alcohol)
DHO: Dipotassium hydrogen orthophosphate
OS: Osteosarcoma
FDA: Food and Drug Administration
TPP: Anionic triphosphate groups
TEM: Transmission electron microscopy
GP: Glycerophosphate
DDA: Degree of deacetylation
NaHCO_3_: Sodium hydrogen carbonate
Chitosan-HTICC: N-[(2-hydroxy-3-trimethylammonium) propyl] chitosan chloride
MSC: Mesenchymal stem cell
LSCM: Laser scanning confocal microscopy
PLGA: Poly (lactic-co-glycolic acid)
kD: Kilo Dalton
MW: Molecular weight
SEM: Scanning electron microscope
CS: Chitosan
TE: Tissue engineering
ECM: Extracellular matrix
*β*-GP: *β*-Glycerophosphate
HPMC*:*: Hydroxypropyl methylcellulose
5-FU*:*: *5-*Fluorouracil.
